# A conversation with Dr. Brett Leav, Vice President of Clinical Development for Public Health Vaccines, Moderna

**DOI:** 10.1017/cts.2022.443

**Published:** 2022-08-19

**Authors:** Kathy Siranosian

**Affiliations:** Clinical Research Forum, Washington, DC, USA

## Top 10 Clinical Research Achievement Awards Q & A

This article is the second in a series of interviews with recipients of Clinical Research Forum’s Top 10 Clinical Research Achievement Awards. In 2021, we honored Pfizer and Moderna with the Clinical Research Forum Award for Extraordinary Impact on Health, to acknowledge the incredible scientific achievement and effort of the many colleagues that have led to the successful development of the Pfizer and Moderna COVID-19 vaccines. The rapid development of these vaccines shows what clinical and translational research can accomplish at its best and is a demonstration of the research continuum from basic science through translation into clinical research to safe and effective vaccines to protect human health. This article is with Dr Brett Leav, Vice President of Clinical Development for Public Health Vaccines from Moderna, who is responsible for the global clinical development of vaccines and led the Moderna COVID-19 vaccine effort.

## What were the early influences that shaped your interest in science and medicine?

For as long as I can remember, I have had an interest in understanding how nature works. My father, who was a veterinarian and an academic scientist, was a big influence, and by the time I was in high school, I realized that I, too, wanted to pursue a career in the biological sciences. Still, my path ended up being a bit circuitous. I went to a liberal arts college and was a history major – but I also fulfilled all the premed course requirements, thinking that I would probably go to medical school, which I did. My studies and experiences as a young person helped shape my worldview that science was not necessarily well pursued unless it was in the service of others.

## How did you become involved in clinical research?

After completing my residency, I practiced internal medicine for three years, taking care of people of all ages, from young adults to end of life. I saw the power of antibiotics to make a difference in the course of illness and that inspired me to pursue a fellowship in infectious diseases. I learned that the immune system could either manage infection in a productive way, by helping get rid of it, or in a nonproductive way, by generating a harmful immune response. Ultimately, that led me to embark on a career to study how vaccines work to preempt infections. To me, the development of vaccines is the most awe-inspiring accomplishment in all of medicine.

## You mentioned your earlier vaccine work. How did this impact your work on the SARS-CoV-2 vaccine?

Since the early 2000s, I’ve been working on a number of different biologics and vaccines that have impacted human health in important ways, for smaller subsets of people. One was a treatment to prevent rabies, which looking back on it, is my second proudest accomplishment, after the SARS-CoV-2 vaccine. This rabies vaccine has had a tremendous impact in countries like India where there are a lot of wild dogs. I have also developed a treatment to prevent recurrent *Clostridioides difficile* infection (CDI), and prior to joining Moderna, I worked on influenza vaccines. All of these products have benefits, but not to the same level and scope of the SARS-CoV-2 vaccine. When I joined Moderna in April 2020, I believed that messenger RNA vaccine technology had promise, but to be honest, I did not expect it to work as well as it did. My basis for comparison was influenza vaccines, which are about 50% effective, and so I was blown away by the effectiveness of the mRNA vaccines against COVID-19 when the results became known in November of 2020.

## Was there anything else that surprised you during development of the SARS-CoV-2 vaccine?

A striking feature of this work was the unprecedented efficiency in how we conducted the clinical research – and how we were able to do so without compromising the quality of the work and while still ensuring that the product was safe and effective. We learned that you do not necessarily need to wait until the end of the step that precedes the step you're working on, that you can glean the important information from that first step at an earlier stage and move on. Of course, it was a very unique time. But I hope that what will emerge from this is that there can be real-time collaborations between regulatory agencies and the developers of medicine and that this can be done in a way that satisfies the requirements for safety and effectiveness. While we were working on the vaccine, we would pick up the phone and have a response from the FDA in days, whereas before that it could have taken weeks to months. Collaboration like that proved so valuable.

## What advice do you have for people beginning their careers in clinical research?

It is important for people working in this field to maintain a sense of awe, to truly understand that what we do in clinical research matters, that it can actually change the arc of human disease in a meaningful way. When I started writing grants to fund my academic research, I would always include at the end how my hope was that the research would result in a therapeutic. Twenty years later, I have seen firsthand how this is true, how each step a researcher takes leads to the next one, and then to the one after that. The development of the SARS-CoV-2 vaccine is a perfect example. It was enabled by our collaborators at NIH and the work that they, and others before them, did. We were standing on the shoulders of giants.

For the SARS-CoV-2 vaccine, the scientific enterprise was able to function at its highest level of efficiency and power because we had the “trifecta” of academia, industry, and the government all working together to achieve something extraordinary, all collaborating for a common goal. None of what we accomplished would have been possible without the participation of those who enrolled in the clinical trials, as well. You have to remember that half of the people in the pivotal trial received a placebo. Many of them were frontline workers, first responders, people working in supermarkets, bus drivers, and so on. Their participation was essential and heroic.

## What continues to motivate you?

What motivates me is that in my life I have seen how the promise of those little advances enables something larger to come to fruition. I truly believe that there’s no foe that we cannot defeat in terms of the diseases that affect human health, as long as we put our minds to it, follow the science, and listen to our health care professionals.

## Outside of clinical research, what other activities do you enjoy? How do these activities impact your work?

One activity that kept me sane through this time was getting outdoors, just going for a walk in the woods. I have always enjoyed experiencing nature; it gives me the opportunity to think and reflect. Also, I could not have done what I did without the unflagging support of my spouse, who literally handed me three meals a day through a little doorway to where I sat in my office for a year and a half. And my children were so understanding! I’m so grateful for the support of my family, which helped me have the personal sense of peace to stay focused.

